# Supplementary material for: The protein kinase Ire1 has a Hac1-independent essential role in iron uptake and virulence of Candida albicans
Source: PLoS Pathog. 2022 Feb 2;18(2):e1010283. doi: 10.1371/journal.ppat.1010283 (PMC8846550; doi:10.1371/journal.ppat.1010283)
Supplement: S1 Fig — (PDF) [file ppat.1010283.s001.pdf]

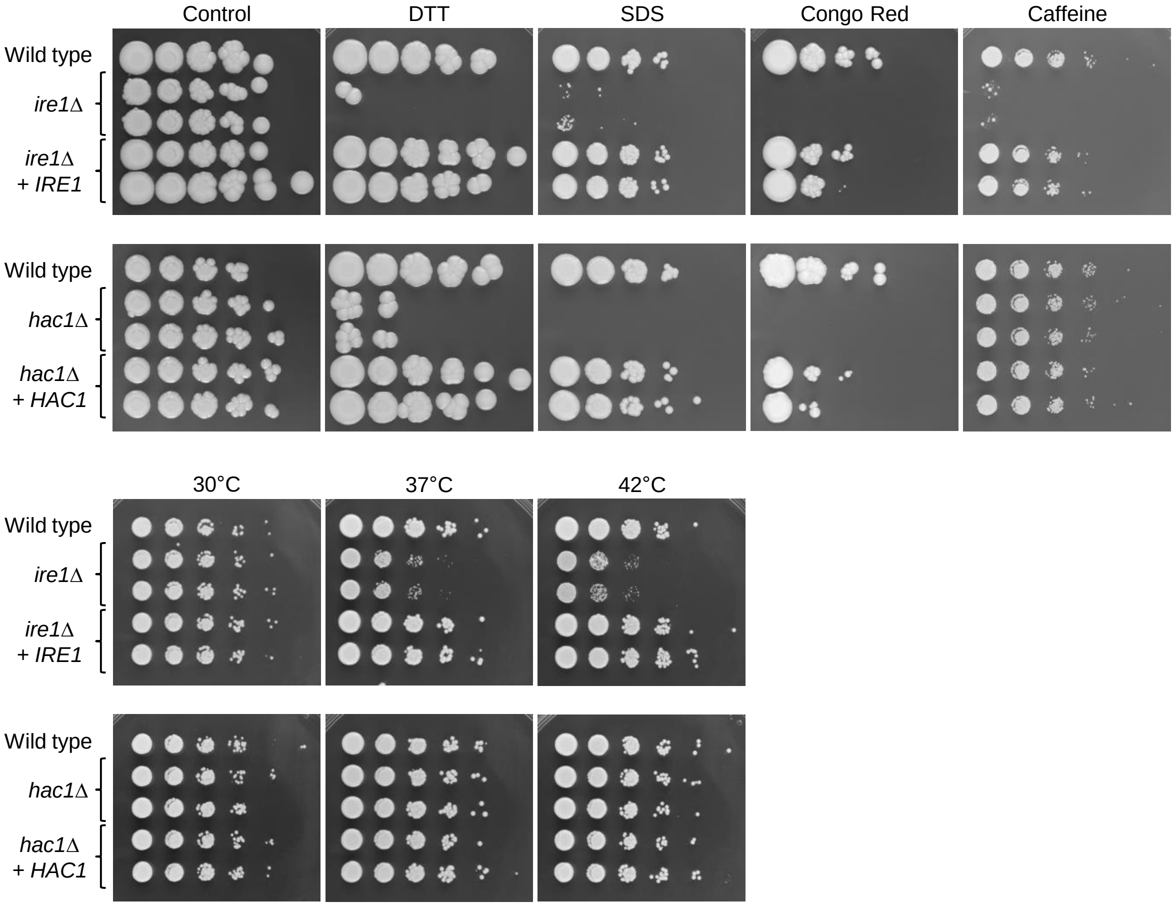

Fig S1. Sensitivity of *ire1*Δ and *hac1*Δ mutants and complemented strains to ER and cell membrane/wall stress and elevated temperatures. YPD overnight cultures of the strains were diluted to an optical density (OD_{600}) of 2.0. Serial 10-fold dilutions were spotted on YPD plates without (control) or with 10 mM DTT, 0.04% SDS, 50 µg/ml Congo Red, or 15 mM caffeine and incubated for 4 days at 30°C. YPD plates were also incubated for 24 h at 30°C, 37°C and 42°C. Both independently generated series of mutants are shown in each case.
