# Supplementary material for: The protein kinase Ire1 has a Hac1-independent essential role in iron uptake and virulence of Candida albicans
Source: PLoS Pathog. 2022 Feb 2;18(2):e1010283. doi: 10.1371/journal.ppat.1010283 (PMC8846550; doi:10.1371/journal.ppat.1010283)
Supplement: S2 Fig — (PDF) [file ppat.1010283.s002.pdf]

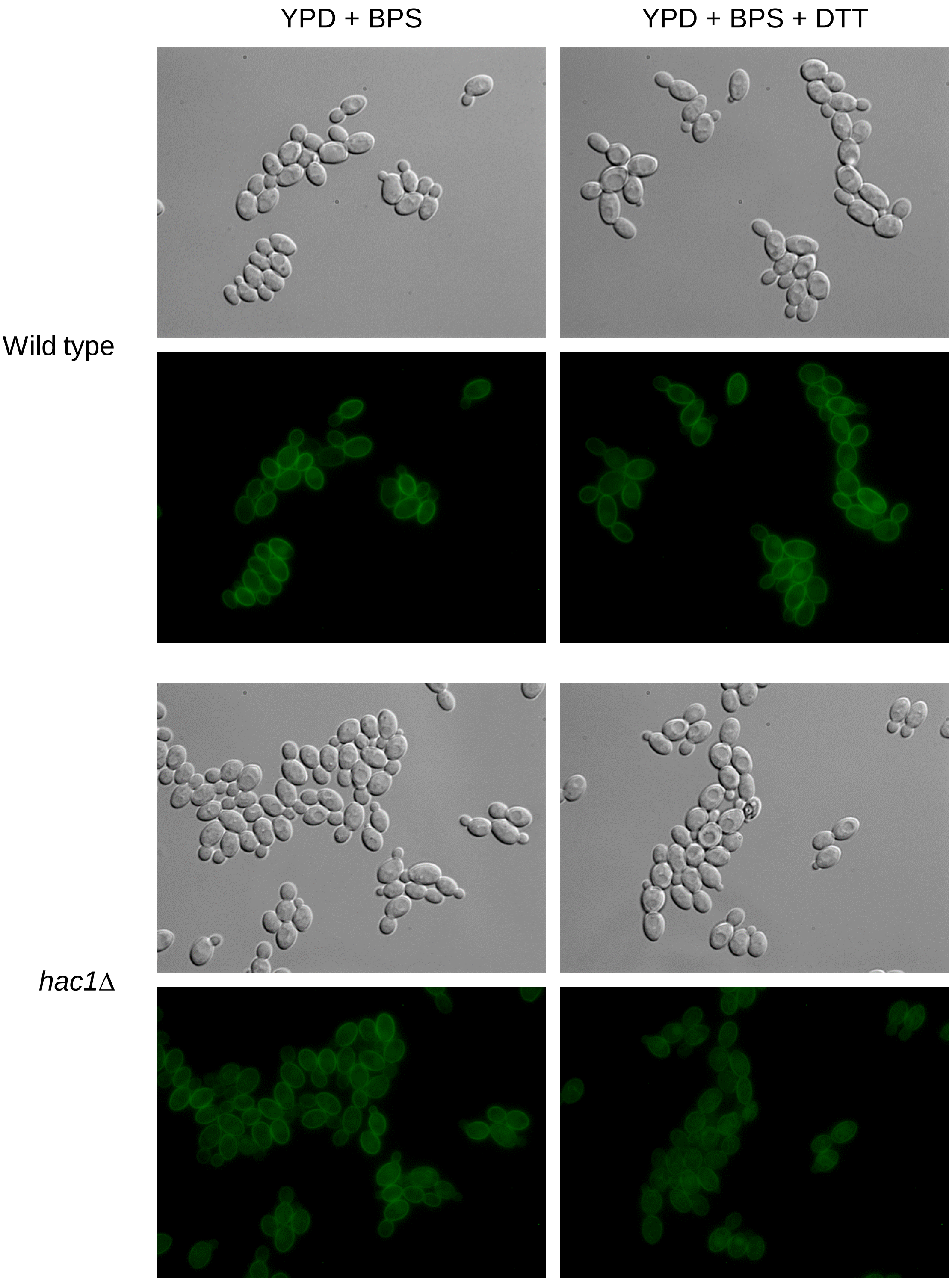

Fig S2. Hac1 contributes to correct Ftr1 localization under conditions of both iron limitation and ER stress. Overnight cultures of wild-type and *hac1Δ* strains containing a *GFP*-tagged *FTR1* allele were inoculated in fresh YPD medium with 500 µM BPS in the absence or presence of 5 mM DTT. After 5 h of incubation at 30°C cells were imaged by DIC and fluorescence microscopy.
