# Supplementary material for: The protein kinase Ire1 has a Hac1-independent essential role in iron uptake and virulence of Candida albicans
Source: PLoS Pathog. 2022 Feb 2;18(2):e1010283. doi: 10.1371/journal.ppat.1010283 (PMC8846550; doi:10.1371/journal.ppat.1010283)
Supplement: S3 Fig — (PDF) [file ppat.1010283.s003.pdf]

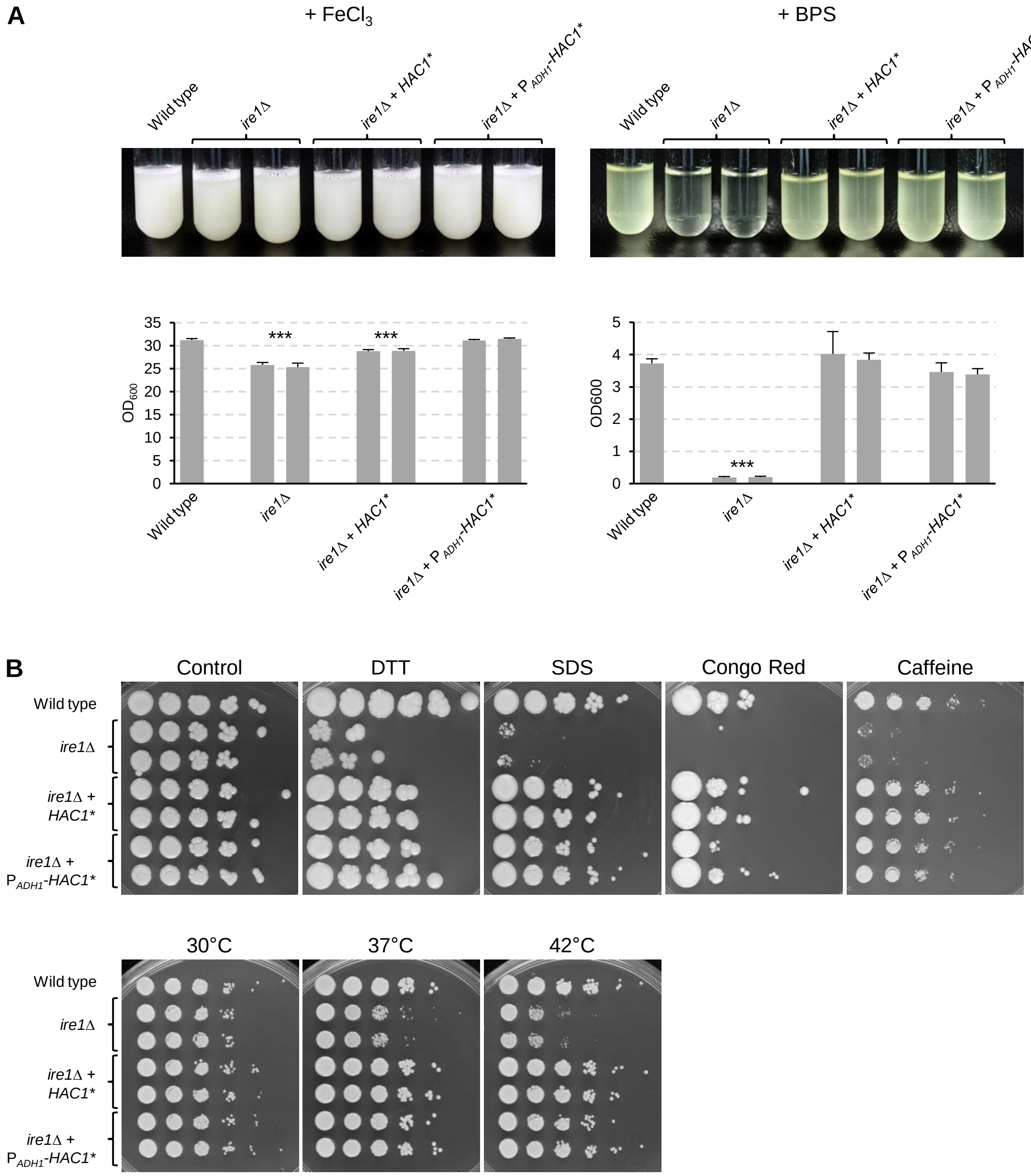


**Fig S3. Removal of the intron in one of the endogenous *HAC1* alleles is sufficient to rescue growth defects of *ire1*Δ mutants.** (A) The wild-type strain SC5314, *ire1*Δ mutants, and derivatives in which one of the endogenous *HAC1* alleles was replaced by the pre-spliced *HAC1** (*ire1*Δ + *HAC1**) or which expressed *HAC1** from the *ADH1* promoter (*ire1*Δ + $P_{ADH1}$-*HAC1**) were grown in minimal medium with (+ $FeCl_3$) or without iron (+ BPS). Cultures were photographed after 4 days of growth at 30°C (top panels) and the optical densities of three independent cultures of each strain determined (bottom panels showing means and standard deviations). Results for both independently generated series of strains are shown separately; data were combined for statistical analysis. Significant differences from the wild type are indicated by stars (***, $p < 0.001$). (B) Overnight cultures of the same strains were serially 10-fold diluted, spotted on YPD plates without (control) or with 10 mM DTT, 0.04% SDS, 50 µg/ml Congo Red, or 15 mM caffeine and incubated for 4 days at 30°C (top panels). The strains were also inubated on YPD plates for 24 h at 30°C, 37°C, and 42°C (bottom panels) Both independently generated series of mutants are shown in (A) and (B).
