# Supplementary material for: The protein kinase Ire1 has a Hac1-independent essential role in iron uptake and virulence of Candida albicans
Source: PLoS Pathog. 2022 Feb 2;18(2):e1010283. doi: 10.1371/journal.ppat.1010283 (PMC8846550; doi:10.1371/journal.ppat.1010283)
Supplement: Attachment — Submitted filename: figR2.pdf [file ppat.1010283.s009.pdf]

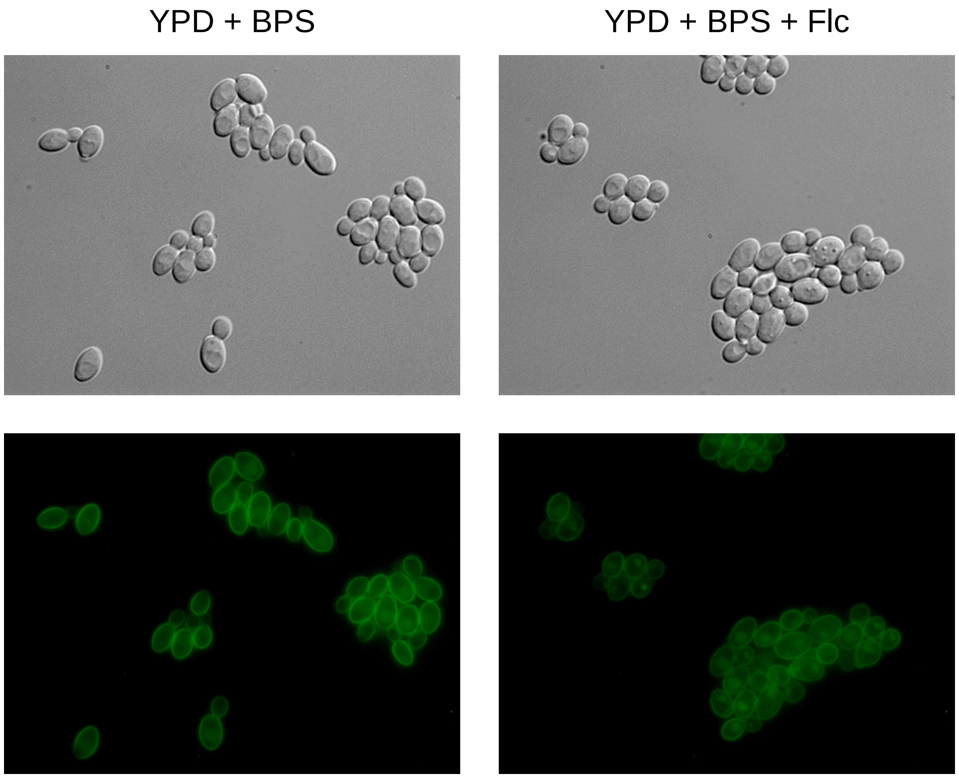

Fig R2. Effect of fluconazole treatment on Ftr1 localization. Overnight cultures of wild-type cells carrying a *GFP*-tagged *FTR1* allele were inoculated in fresh YPD medium with 500 µM BPS in the absence or presence of 10 µg/ml fluconazole (Flc). After 5 h of incubation at 30°C cells were imaged by DIC and fluorescence microscopy.
