# Supplementary material for: The protein kinase Ire1 has a Hac1-independent essential role in iron uptake and virulence of Candida albicans
Source: PLoS Pathog. 2022 Feb 2;18(2):e1010283. doi: 10.1371/journal.ppat.1010283 (PMC8846550; doi:10.1371/journal.ppat.1010283)
Supplement: Attachment — Submitted filename: Responses to the review comments.docx [file ppat.1010283.s007.docx]

**Responses to the reviewers’ comments**

Our responses are highlighted in blue for clarity.

**Part I – Summary**

Reviewer #1:

This is an interesting analysis of the contribution of the Ire1 UPR sensor to the biology and virulence of Candida albicans, focusing on the role of Ire1 in growth under Fe-limiting conditions. Using a protein kinase deletion mutant library, the Ire1 transmembrane kinase was found to be essential for growth in Fe-depleted media, but surprisingly independent of the Sef1 transcription factor involved in the adaptation to low Fe. The results demonstrate that the high-affinity plasma membrane Fe permease Ftr1 is mislocalized to the ER instead of the plasma membrane in the ire1 mutant. This was not a generalized effect on nutrient transporters since Mep2 was unaffected by ire1 deletion. Interestingly, Fe limitation did not enhance ER stress signaling from the Ire1 sensor, and the gene encoding the Hac1 transcription factor that is activated by Ire1 was dispensable for the movement of Ftr1 to the plasma membrane. However, expression of a pre-spliced copy of HAC1 mRNA in the ire1 mutant bypassed the need for Ire1 with respect to proper localization of Ftr1 and growth in low Fe. Finally, the ire1 and hac1 mutants were avirulent, indicating that C. albicans, like many other pathogenic fungi, rely heavily on ER stress responses in the host environment.

This is an interesting body of work and the logical flow of the data presentation made the manuscript a pleasure to read. The major strength is that the study uncovers a novel aspect of UPR function that involves the localization of a high-affinity Fe permease to the plasma membrane, providing an explanation for how this pathway contributes to the adaptive response to low Fe conditions. The major weakness is that the interpretation of the data in the context of Ire1 having hac1-independent functions in this aspect of Fe homeostasis needs some clarification.

See our response to the specific comment (part III).

Reviewer #2:

Ramírez-Zavala et al. generated and employed a deletion mutant library for protein kinase genes in Candida albicans to identify a role for the Ire1 kinase in growth upon iron deprivation. The activity of Ire1 is independent of the iron regulator Sef1 and instead influences localization of iron uptake functions. These activities are independent of the transcription factor Hac1 that is activated by Ire1 although, intriguingly, expression of a pre-spliced version of Hac1 rescued the iron related phenotypes of the ire1 mutant. Importantly, Ire1 and Hac1 are required for systemic candidiasis. The study nicely builds on previous interesting findings on the connection between the Ssn3 kinase and the iron regulator Sef1, and provides novel new information on iron regulation and Ire1 in Candida albicans. The data are clearly presented and the manuscript is well written.

Reviewer #3:

The major finding of the study is that the protein kinase Ire1 has a role for C. albicans growth under iron depletion condition and is required for virulence. However, this is recently published (ref. #37. Figure 5 showed growth defect of an ire1 mutant on YPD+BPS and reduced virulence).

We wish to emphasize that we had uncovered the requirement of Ire1 for growth under iron-limiting conditions long before the recent study by Sircaik *et al*. was published. These authors only speculated about a possible reason for the observed growth defect of an *ire1* knockdown mutant on iron-depleted medium, and otherwise focused their study on confirming the conserved role of Ire1 in the UPR of *C. albicans*. In contrast, we went on with further detailed investigations and elucidated the underlying mechanism of the growth defect of cells lacking Ire1 under iron-limiting conditions and the relationship with Hac1 function. As pointed out in our paper, that *ire1* mutants would exhibit reduced virulence was to be expected, considering their sensitivity to ER stress and the cell wall and morphogenesis defects that were already described by Blankenship *et al*. in 2010. However, we investigated potential differences in virulence attenuation of *ire1*Δ and *hac1*Δ mutants and whether activation of Hac1 would overcome the expected virulence defect of *ire1*Δ mutants.

This study also concludes that iron limitation does not cause increased endoplasmic reticulum stress because HAC1 intron removal was not observed under iron depletion condition. Furthermore, the hac1 mutant was able to grow under the iron depletion condition. But Ire1 is known to also mediate other ER membrane stresses that are independent of Hac1. Whether and how iron depletion affects ER membrane stress needs further experiments.

Many researchers use *HAC1* mRNA splicing as the readout for ER stress, and so did we. Some researchers also detected Ire1 clustering as a response to ER stress, for example in the study referred to by the reviewer (Cohen *et al*. 2017) in the specific comment (part II). Ire1 clustering is thought to serve as a platform for *HAC1* mRNA recruitment and subsequent splicing. It should be pointed out that Cohen *et al*., using this alternative readout, also found that iron limitation by itself did not cause ER stress: “From these findings, we conclude that iron depletion or repletion does not, in itself, cause ER stress”. Note that Ire1 does not mediate ER membrane stresses; to the contrary, Ire1 overcomes ER stresses, by mediating an appropriate reaction (but we assume that this was a typing error in the reviewer’s comment).

Another major finding of this study is that Ire1 is required for the plasma membrane localization of the iron permease Ftr1 under iron depletion condition. This has been used as a read out for Hac1-independent Ire1 activation in this study. This is a new observation, but it is not clear how Ire1 is regulated by iron depletion. Are there other downstream effectors of Ire1 that contribute to growth under low iron conditions?

As explained in response to a similar comment by reviewer 1 (see below, part 3) and in the revised manuscript (lines 326-331 and 428-437), we hypothesize that chaperones that ensure proper association of Ire1 with the ferroxidase and cotransport of the complex are reasonable candidates for Ire1 downstream effectors that are required even under non-stressed conditions, especially when there is a high demand for the high-affinity iron permease under iron-limiting conditions. We do not claim that iron depletion regulates Ire1 (see also our response to the previous comment); it may simply be the basal (Hac1-independent) activity of Ire1 that is required for proper transport of the high-affinity iron permease.

**Part II – Major Issues: Key Experiments Required for Acceptance**

Reviewer #1: None

Reviewer #2: None noted

Reviewer #3:

1. This study does not have any experiments that can directly link iron depletion to Ire1 activation. Because HAC1 intron is not removed upon iron depletion, this study concluded that iron depletion does not lead to the ER membrane stress. This conclusion is questionable based on literature. Ire1 affects azole susceptibility in C. albicans (ref. 37) and S. cerevisiae (PMCID: PMC5665437). The S. cerevisiae paper showed a cross-talk between the ER stress signaling and iron/heme homeostasis. Based on the S. cerevisiae study, there are several experiments to determine if similar cross-talk exists in C. albicans. For example, does Sef1 translocate to the nucleus upon UPR activation by DTT. Does heme limitation affect Ftr1 localization to the PM? Is sterol biosynthesis required for Ftr1 PM localization?

As already pointed out in our response to the second comment by this reviewer in part I, *HAC1* mRNA splicing is used by many researchers as a readout for ER stress and Ire1 activation, and we did not find an effect of iron depletion on Ire1 activation, which is in line with the results reported by Cohen *et al*. for *S. cerevisiae* (PMC5665437). The subsequent arguments by the reviewer lack logic and are not entirely correct. Azoles inhibit ergosterol biosynthesis and therefore membrane composition and functionality. This may cause membrane stress and Ire1 activation irrespective of iron availability, but also impede Ire1 clustering in response to ER stress. Cohen *et al*. did not perform azole susceptibility tests with *S. cerevisiae ire1* mutants. However, they assessed the effect of ergosterol biosynthesis inhibitors on Ire1 clustering and did not observe an effect in the absence of ER stress (similarly, Sircaik *et al*. found that fluconazole treatment did not cause *HAC1* mRNA splicing). Notably, the inhibitors reduced Ire1 clustering in response to ER stress caused by DTT treatment, demonstrating that inhibition of ergosterol biosynthesis actually impedes the UPR.

Concerning the experiments suggested by the reviewer, we note the following:

Wimalasena *et al*. (2008) analyzed the transcriptional response of *C. albicans* to ER stress caused by exposure to DTT and tunicamycin and did not observe upregulation of iron limitation-induced genes (which is now known to depend on Sef1). Based on these published findings, there is no rationale to expect Sef1 translocation to the nucleus upon UPR activation by DTT. Since the reviewer requested this experiment, we constructed strains containing a *GFP*-tagged *SEF1* allele and observed Sef1 localization under different conditions. As expected, Sef1 was upregulated and translocated to the nucleus when the cells were grown in the presence of the iron chelator BPS, but not upon UPR induction by treatment with DTT. These results are shown in a supplemental figure (Fig R1) that is meant for review purposes only. We did not include the experiment in the paper because it will only confuse readers. As explained above, based on the results reported by Wimalasena *et al*. there was no logic to perform the experiment. Furthermore, it detracts from the aim of our investigation, which was not an analysis of the role of Ire1 in the UPR in *C. albicans*.

If heme limitation affects Ftr1 localization to the membrane, this would be an effect of impaired heme-dependent processes, such as ergosterol biosynthesis, which has been shown Cohen *et al*. to negatively impact Ire1 function. Therefore, testing this would not add to our paper, and it does not make sense to construct additional mutants lacking genes required for heme biosynthesis (used by Cohen *et al*. for this purpose in *S. cerevisiae*) and probing them for Ire1 functionality.

It can of course be expected that sterol biosynthesis is required for Ftr1 localization to the plasma membrane, because sterols are essential membrane components. We are not aware of viable *C. albicans* mutants that do not synthesize sterols at all. However, we suspected that the reviewer in fact may have wondered if inhibition of ergosterol biosynthesis (e.g. by azoles), which results in altered sterol composition of membranes, affects Ftr1 localization. We tested if treatment of the cells with fluconazole would affect Ftr1 localization and found that Ftr1 was still mainly localized at the plasma membrane, although some intracellular protein was also detected under azole stress. This result is shown in a supplemental figure (Fig R2) that is meant for review purposes only, because the aim of our study was not to investigate the importance of sterols for cellular functions in *C. albicans*.

2. Why can a pre-spliced HAC1 bypass the requirement of Ire1 for Ftr1 PM localization and for growth under iron depleted conditions when Hac1 is not required for either. This data would suggest that the iron uptake is not completely Hac1 independent. Why the pre-spliced HAC1 completely bypassed the ire1 requirement for growth under iron depleted conditions in vitro, but did not fully bypassed the virulence defect of the ire1 in vivo?

For explanations of why a pre-spliced *HAC1* can bypass the requirement of Ire1 for Ftr1 localization and growth under iron-depleted conditions, although Hac1 is dispensable for these phenotypes in wild-type cells, and why the pre-spliced *HAC1* did not fully restore virulence of *ire1*Δ mutants, see our responses to the first comment by reviewer 1 and the last comment by reviewer 2, respectively, in part III. The suggestion that iron uptake is not completely Hac1 independent does not make sense to us. Phenotypic analysis of a specific gene deletion mutant is the gold standard for assessing the importance of the encoded protein for that phenotype, and our *hac1*Δ mutants exhibited wild-type growth in iron-depleted medium in our experiments. Of course, Hac1 might be required for iron uptake under conditions in which Ire1 functionality is disturbed in some way, as we demonstrate with the strains that contain a constitutively activated Hac1 in the absence of Ire1.

3. The ire1 mutant seemed to have reduced transcription levels of FTR1 and FET31 (Fig. 5A). This is consistent with transcriptional profiling of WT and ire1 by Sircaik et al (PMCID: PMC5665437). But the authors concluded no change? Does the ire1 mutant affect Sef1 nuclear localization under iron depletion condition?

This comment contains several incorrect statements. When discussing our findings, we had pointed out already in the original manuscript that the mRNA levels of iron limitation-induced genes were slightly lower in the *ire1*Δ mutants than in the wild type after 5 h of growth in iron-depleted medium, and explained that this was likely due to the growth defect of the *ire1*Δ mutants in this medium, which results in lower amounts of newly produced mRNAs compared to those of constitutively expressed genes such as the *ACT1* control (lines 448-453 in the revised manuscript). The important point is that all tested genes were strongly upregulated upon iron limitation also in the *ire1*Δ mutants. This result is not consistent with the findings by Sircaik *et al*. (PMC5665437 is not the PMCID for this paper). These authors compared the transcriptional profiles of wild-type cells and *ire1* mutants under iron-replete conditions and did not test their expression under iron-limiting, inducing conditions. The upregulation of the tested iron limitation-induced genes depends on Sef1, as has been shown by Chen *et al*. (2011). Since these genes were strongly induced in the *ire1*Δ mutants, it did not make sense to verify that Sef1 is localized to the nucleus under iron-depletion conditions in the *ire1*Δ mutants - how else should the transcription factor activate its target genes? Nevertheless, since we now generated a GFP-tagged *SEF1* allele (see response to comment 1 above), we also introduced it into the *ire1*Δ mutants. As expected, Sef1 was normally upregulated and localized to the nucleus in the absence of Ire1. This result is shown in a supplemental figure (Fig R3), which is meant for review purposes only, because Fig. 5 already shows that Sef1 target genes were upregulated in response to iron depletion in the *ire1*Δ mutants. Since we did not generate derivatives of the *mec1*Δ and *vps15*Δ mutants containing the GFP-tagged *SEF1* allele, the result would also not fit into Fig. 5, in which we still analyzed all three kinase deletion mutants.

4. The introduction provides no background on Ire1. The result starts with several figures that basically show negative data or data not related to Ire1. Most of these could go into supplemental material. The paper (almost half of the content) starts with experiments that do not contribute to our understanding of how iron depletion regulates Ire1. Real data starts from line 224. The manuscript also missed some key papers, such as PMCID: PMC5665437 and PMCID: PMC7319344.

We wish to emphasize that the goal of our paper at the outset was not to study the function of Ire1 in *C. albicans*, but to test whether an activated form of Sef1 would overcome its reported dependence on Ssn3, in order to gain more insight into how *C. albicans* adapts to iron limitation. The task of the introduction of a paper is to explain the rationale of the study and to provide the necessary background knowledge, and this is what we have done. Ire1 came into play only after the screening of our protein kinase deletion mutant library (after we unexpectedly found that Ssn3 was not required for Sef1 function and growth under iron-limiting conditions), and we always provide the necessary information about its functions at appropriate places. We reject the proposal to move the initial results (including the dispensability of Ssn3 for Sef1 function and growth under iron-limiting conditions, the construction and screening of a comprehensive protein kinase deletion library, the growth assays with the *ire1*Δ and other kinase mutants, tests for Sef1 functionality in these mutants and their ability to induce the expression of Sef1-dependent genes in response to iron limitation) to supplemental material, since these contain important information and form the basis of the remainder of the paper (see also the other reviewers’ opinions “…the logical flow of the data presentation made the manuscript a pleasure to read” by reviewer 1 and “The data are clearly presented and the manuscript is well written” by reviewer 2, which we have included above for this purpose). The contention that these results are not real data is unacceptable. It should be noted again that we do not claim that iron depletion regulates Ire1 (see also our response to the second comment by this reviewer in part I). In the revised manuscript, we have now included a reference to the paper by Cohen *et al*. (PMC5565437), as suggested by the reviewer (lines 414-416). The results of that paper confirm (for *S. cerevisiae*) our finding that iron limitation by itself does not cause ER stress. Only under conditions of ER stress (which we did not study and included only as control in some experiments) did Cohen *et al*. observe an effect of iron limitation on the UPR, and this was caused by the fact that some ergosterol biosynthesis enzymes (as well as many other proteins) require heme iron, which presumably affected membrane composition and Ire1 clustering in response to ER stress. The paper by Alkafeef *et al*. (PMC7319344) studied the function of the monothiol glutaredoxin Grx3 in the regulation of iron-dependent processes, but we did not see how this relates to our present investigation (e.g., neither Ire1 nor Ftr1 are even mentioned in that paper). The reviewer did not provide a reason for this suggestion.

**Part III – Minor Issues: Editorial and Data Presentation Modifications**

Reviewer #1:

There was one aspect that I did not fully understand. The ire1 deletion mutant is defective in Ftr localization & growth in low Fe, which could be rescued by expression of spliced hac1, supporting a role for the Hac1 transcription factor in this process. Since the hac1 deletion mutant did not show this defect, the data are interpreted as evidence that Ire1 has hac1-independent functions in controlling this process (though presumably via different mechanisms). It seems to me that the ability of the processed hac1 to complement the Ftr localization defect in deltaire1 does support a role for Hac1 in this process, which is novel and important. However, I am less convinced that the normal Ftr localization observed in deltahac1 (where ire1 is present but unable to trigger the canonical pathway) necessarily indicates that it is a hac1-independent function of ire1 that is responsible for controlling Ftr localization. My interpretation of the data is that something else is clearly able to ensure proper localization of Ftr and restore growth in low Fe when hac1 is deleted, but this does not necessarily mean that Ire1 is the relevant protein. The data do raise the interesting possibility of involvement of hac1-independent functions of Ire1 playing a role, but other possibilities could also account for this result. This could be addressed by softening the Ire1 conclusion or, if I have misinterpreted the data, clarifying the evidence that clearly supports a role for Ire1 outside of the canonical pathway.

Our interpretation of these results is as follows. Ire1 is also required for full functionality of the secretory pathway in the absence of detectable ER stress, conditions in which Hac1 is dispensable. Ire1 may ensure the optimal functionality of chaperones or other proteins that are required for the association and cotransport of the Ftr1-ferroxidase complex. The upregulation of these proteins that is caused by the activated Hac1 would bypass the requirement for Ire1. It is also possible that Ire1 directly interacts with Ftr1 and/or the ferroxidase to help in proper folding/association, and that this function is bypassed by Hac1-induced chaperone overexpression. We have explained this in more detail in the revised manuscript (lines 326-331 and 428-437). Whatever the precise mechanism is, our results clearly show that Ftr1 localization depends on Ire1 but not on Hac1 in the absence of ER stress. We reasoned that the basal function of Ire1 might not be sufficient and require Hac1 for proper Ftr1 transport under conditions of ER stress. Therefore, we observed Ftr1 localization under conditons of both iron limitation and ER stress (lines 325-337). As shown in the new S2 Fig, Ftr1 was partially retained in the ER in the *hac1*Δ mutants when cells grown in iron-depleted medium were additionally treated with DTT. The Ftr1-GFP signal was relatively weak under these conditions in the *hac1*Δ mutants, but the result supports this model.

A prior study reported the inability to obtain a complete deletion mutant of Ire1 in C. albicans (Blankenship, PlosPathogens 2010), which prompted a recent study to undertake an ire1 knockdown strategy instead of attempting a homozygous deletion (Sircaik et al.,Cell Microbiol. 2021). I would be curious about the authors thoughts on this apparent difference in essentiality. Does this deletion mutant filament?

It should be noted that Blankenship *et al*. generated a homozygous *ire1* transposon insertion mutant, which is described throughout that study as a null mutant, but the authors make a cautionary statement that the insertion was relatively distal and that they failed to obtain a full deletion mutant. Nevertheless, the insertion mutants used in Blankenship *et al*. were highly sensitive to cell wall stress and defective in filamentation and biofilm formation. We note that Sircaik *et al*. ignore these previous findings and report the same phenotypes of *ire1* knock-down mutants (obtained from the Mitchell lab!) as novel results of their study. We had also observed a filamentation defect (as well as sensitivity to cell wall stress) of our *ire1*Δ mutants in other phenotypic screenings of our protein kinase deletion mutant library, but do not describe these findings in our paper, because they just confirm the previous results by Blankenship *et al*. (which are mentioned) and are not directly related to our present investigation.

Reviewer #2:

Line 75. The wording is funny here because it sounds like iron forms a functional permease rather than being the transported substrate.

The meaning of the sentence was “Ftr1 associates with one of several redundant multicopper ferroxidases...to form a functional iron permease”, as indicated by the comma inserted after Ftr1 in the original sentence. To avoid misunderstanding, we have split this sentence into two sentences: “Ftr1 associates with one of several redundant multicopper ferroxidases to form a functional iron permease. The ferroxidase reoxidizes reduced Fe^2+^, generated by cell membrane-located iron reductases, and directly channels the produced Fe^3+^ to Ftr1” (lines 73-75).

Line 117. It would help the reader if the SEF1-GAD allele (artificially activated, line 489) was described in more detail here. The activated version of Sef1 is used extensively in the following experiments and it is therefore important to describe its properties thoroughly when it is first introduced.

The library of artificially activated zinc cluster transcription factors, including Sef1, was already described in the introduction. We have modified this part as follows: “In a previous study from our lab we had used a library of artificially activated forms of all *C. albicans* zinc cluster transcription factors, generated by fusing the Gal4 activation domain (GAD) to their C-terminus, to uncover their biological roles and reveal novel phenotypes conferred by gain-of-function mutations in these transcriptional regulators (15). Strains expressing the hyperactive *SEF1-GAD* allele produced a yellow culture supernatant...” (lines 98-104). In this way, the meaning of *SEF1-GAD* and the key feature of the artificially activated Sef1 should be clear from the beginning.

Line 125, Fig. 1A, B. The strains grow quite poorly on the medium with BPS – especially the ssn3 mutant with the two versions of SEF1. Could it be that the poor growth makes it difficult to evaluate riboflavin production for the ssn3 mutant? A similar question arises for the information on lines 180-181 and the conclusion that no riboflavin is produced by the kinase mutants even though they are growing quite poorly or not at all. Also, is there an explanation for the growth impact of SEF1, particularly in the ssn3 mutant background +BPS in Fig. 1B?.

It is conceivable (and often observed) that constitutive overexpression of a transcription factor, and even more so of a hyperactive form, can have a negative impact on the fitness of the cells. This was also the case for *SEF1*, and we have pointed this out in the text (lines 124-126). That the negative impact of *SEF1* overexpression was somewhat more pronounced in the *ssn3*Δ mutants than in the wild type, and further increased under stress conditions (iron depletion) in both strain backgrounds, may relate to the fact that Ssn3, as part of the mediator complex, has a broad role in transcriptional regulation and cellular activities. Therefore, *ssn3*Δ mutants may be more sensitive to transcription factor overexpression than wild-type cells. Nevertheless, *SEF1* overexpression did not detectably disturb riboflavin production by the *ssn3*Δ mutants under iron-limiting conditions (Fig. 1A). Note also that the *ssn3*Δ mutants without overexpressed *SEF1* grew as well as the wild type in the iron-depleted medium and there was no visible difference in riboflavin production. We did not find mutants in our library that were defective in riboflavin production despite growth in iron-depleted medium, i.e., absence of riboflavin was always simply due to the fact that the mutants did not grow in that medium (no cells, no riboflavin production). This is what we pointed out in the sentence referred to by the reviewer. The *ire1*Δ, *mec1*Δ, and *vps15*Δ mutants did not detectably produce riboflavin because (not even though) they did not grow in iron-depleted medium (see lines 180-182 in the revised manuscript).

Of course, this study would be stronger if more insights were obtained into the Hac1-independent roles of Ire1. I wonder if the authors might speculate more about potential targets. For example, are there clues from the cited study of Sircaik et al?

See our response to the first comment by reviewer 1 above (part III). The study by Sircaik *et al*. does not provide clues, because they did not try to identify Ire1 interaction partners.

I was intrigued that the pre-spliced version of Hac1 did not restore full virulence to the ire1 mutant. The authors indicate that statistical analysis did not support a difference but it seems that the authors could speculate more about this intermediate result.

There is a straightforward explanation for the slightly decreased virulence of these strains. The stress conditions encountered during an infection may require complete conversion of the *HAC1* mRNA into its spliced form, at least in some environments. This is not achievable in the *ire1*Δ mutants in which one of the two *HAC1* alleles is pre-spliced, but the mRNA produced from the other allele cannot be spliced due to the absence of Ire1. Therefore, these cells might have lower levels of active Hac1 than a wild-type strain. We have added these considerations in the revised manuscript (lines 365-370). One could possibly confirm this hypothesis by constructing and testing strains in which both *HAC1* alleles are pre-spliced, but we discarded this option for two reasons: The difference in virulence between *ire1*Δ mutants containing the pre-spliced *HAC1* and the wild type was small (not even statistically significant), so that sacrificing additional animals does not seem justifiable. Furthermore, the constitutive expression of maximal levels of activated *HAC1* may not be desirable for the cells throughout an infection and by itself have a negative impact on their fitness. If virulence were still incompletely restored, we could not be sure why.
